# Differential Uptake and Utilization of Two Forms of Nitrogen in *Japonica* Rice Cultivars From North-Eastern China

**DOI:** 10.3389/fpls.2019.01061

**Published:** 2019-09-04

**Authors:** Jun Yi, Jiping Gao, Wenzhong Zhang, Chen Zhao, Yan Wang, Xiaoxi Zhen

**Affiliations:** Rice Research Institute of Shenyang Agricultural University, Key Laboratory of Northern Japonica Rice Genetics and Breeding, Ministry of Education and Liaoning Province, Key Laboratory of Northeast Rice Biology and Genetics and Breeding, Ministry of Agriculture, Shenyang, China

**Keywords:** *japonica* rice, nitrogen metabolism, nitrogen use efficiency, grain yield, panicle

## Abstract

*Japonica* rice is widely planted in north-eastern China because of its superior food quality and stable grain yields. Nitrogen (N) is an essential element for rice growth, and development and its availability directly impacts on rice yields. The knowledge of N uptake and its utilization characteristics in *japonica* are thus important areas of research. Three *japonica* rice cultivars, SN265, SN1401, and SN9816, which are planted across large areas of north-eastern China, were used here to evaluate the uptake and utilization along the life cycle of both ammonium $\left(NH_{4}^{+}\right)$ and nitrate $\left(NO_{3}^{-}\right)$ in hydroponically grown plants. The plants were grown in one of three different solutions with varying $NH_{4}^{+}$:$NO_{3}^{-}$ ratios: 1:0, 0:1, and 1:1 (The total N content was 40 mg L^−1^ for each treatment). At the tillering stage, when only $NO_{3}^{-}$ was provided, lower rates of N uptake and enzyme activities of three rice plants resulted in reduced tiller numbers. During the reproductive stage, the $NH_{4}^{+}$ and $\left(NH_{4}^{+}\right)$ uptake rates in SN1401 were consistently maintained at high levels, whereas the rates in SN265 and SN9816 were significantly lower, across all three treatments. At the booting stage, when only $NO_{3}^{-}$ was provided, SN1401 plants had significantly higher expression levels of *OsNRT2.1* and *OsNRT2.2*, higher activity of nitrate reductase in the roots, and higher activity levels of glutamine synthetase and glutamate synthase in the leaves, compared with the SN265 and SN9816 plants. The higher enzyme activity was beneficial to the secondary assimilation of N, which ultimately promoted panicle development in SN1401. Consequently, the grain yield per plant of SN1401 was the highest with solutions of both $NH_{4}^{+}$ and $NO_{3}^{-}$. These results indicate that selecting a rice cultivar with higher utilization of $NO_{3}^{-}$ is beneficial for increasing the number of grains per panicle, grain yield, and N use efficiency.

## Introduction

Rice (*Oryza sativa* L.), is one of the most important global food crops and is widely cultivated in Asia, with *japonica* being a popular subspecies. In China, *japonica* rice is widely planted in the north-eastern and Jianghuai regions. In recent years, because of its food quality and increasing market demand by consumers, *japonica* rice production and cultivation have been gaining more attention. In plant growth and development, nitrogen (N) is one of the most liming mineral nutrients for crop production, and the forms of N that are available for plant roots to absorb from the soil are primarily nitrates, ammonium salts, and amino acids (Wang et al., 1993). In *O. sativa*, *indica* rice cultivars usually have greater nitrate $\left(NO_{3}^{-}\right)$ uptake abilities than the *japonica* rice cultivars (Hu et al., 2015). In addition, different genotypes of *japonica* rice cultivars have been found to have different N uptake rates, grain yields, and N accumulation levels, under the same N treatments in field experiments (Zhang et al., 2007). The need of increasing the N use efficiency to increase rice production has been a long-standing problem for *japonica* rice cultivation and is complicated due to the variability in characteristics of N uptake among the different *japonica* rice cultivars (Koutroubas and Ntanos, 2003).

In rice paddy fields, long-term flooding conditions inhibit the process of nitrification by soil microbes, which results from anaerobic conditions, resulting in high concentrations of ammonium $\left(NH_{4}^{+}\right)$ as the main form of N (Kronzucker et al., 1998). However, under the flooded conditions, rice plants can transfer O_2_ from their shoots to their roots and release it to the rhizosphere *via* special aerated tissue cells. The aerobic conditions allows soil microbial nitrification to occur and produce $NO_{3}^{-}$ in the rice plant rhizospheres for uptake and utilization by the plant (Kronzucker et al., 2000; Kirk and Kronzucker, 2005; Li et al., 2008). In plants, the assimilation pathway of $NH_{4}^{+}$ begins with the uptake of $NH_{4}^{+}$ from the soil into the plant cells by ammonium transporters, which is incorporated to the synthesis of glutamine, through the glutamine synthetase (GS)/glutamate synthase (GOGAT) cycle (Lea and Miflin, 2003). Glutamate dehydrogenase (GDH) catalyzes the other $NH_{4}^{+}$-assimilation pathway by synthesizing glutamic acid from alfa-ketoglutarate (Sonoda et al., 2003). On the other side, the process of $NO_{3}^{-}$ assimilation begins after $NO_{3}^{-}$ uptake by $NO_{3}^{-}$ transporters, followed by the reduction of $NO_{3}^{-}$ to $NO_{2}^{-}$ by nitrate reductase (NR) in the cytoplasm (Lea and Miflin, 1974). Subsequently in plastids, $NO_{2}^{-}$ is reduced to $NH_{4}^{+}$ by nitrite reductase (NIR) (Xuan et al., 2017). The genes of these key N metabolism enzymes play an important role in rice plant growth and also affect the rice yield components (Tamura et al., 2010; Funayama et al., 2013). In a transgenic rice experiment, mutants lacking *OsNADH-GOGAT2* had significantly lower in yields and biomasses, compared with those of their wild-type counterparts (Tamura et al., 2011). In a gene knock-out study of *OsGS1;1* (one of the glutamine synthetase genes encoding for cytoplasmic isoform in rice), the growth rate and the degree of grain filling of the transgenic rice plants was significantly reduced (Tabuchi et al., 2005).

Although the uptake and utilization of different forms of N have been investigated in rice cultivars from southern China, limited information is available on the response of $NO_{3}^{-}$ uptake and its utilization in plants grown in conditions where only $NO_{3}^{-}$ is present. Most researchers have primarily investigated the differences between $NO_{3}^{-}$ and $NH_{4}^{+}$ uptake in rice at the seedling stage, or the effects of greater $NO_{3}^{-}$ concentrations in $NH_{4}^{+}$ nutrient solutions on the processes of N uptake, utilization, and growth for rice (Yan et al., 2011). However, the uptake and utilization of $NO_{3}^{-}$ can be affected by the presence of $NH_{4}^{+}$ in the same nutrient solution (Zhang et al., 2011). For this paper, we have investigated the differences in N uptake and utilization for three common *japonica* cultivars (SN265, SN1401, and SN9816) from north-eastern China with differing N use efficiencies. We have examined the plant responses to hydroponic fertilizer solutions containing $NO_{3}^{-}$ and/or $NH_{4}^{+}$ as the N source, throughout multiple plant growth stages. The results in this study showed that the lower rates of $NO_{3}^{-}$ uptake and enzyme activities of three rice plants resulted in reduced tiller numbers at the tillering stage. During the reproductive stage, the $NH_{4}^{+}$ and $NO_{3}^{-}$ uptake rates in SN1401 were consistently maintained at high levels, whereas the rates in SN265 and SN9816 were significantly lower, across all three treatments, which also resulted in the higher grain yield in SN1401. These results provided some evidence for the N management in different growth stages of rice in the field agriculture.

## Materials and Methods

### Plant Materials

Three *japonica* rice cultivars, SN265, SN1401, and SN9816, were chosen based on their different responses to N applications in field trials (location: 41°48′ N, 123°25′ E) of 30 *japonica* rice cultivars carried out in 2015. Their agronomic traits are shown in **Supplementary Table 1**.

### N Treatments and Growth Conditions

The primary nutrient solution for the hydroponically grown plants was prepared according to a formula from the International Rice Research Institute (IRRI) (Yoshida et al., 1976). The conventional nutrient solution from the IRRI was modified to create the three different conditions for our treatments, by varying the concentration ratios of $NH_{4}^{+}$ and $NO_{3}^{-}$: 1:0, 0:1, and 1:1, respectively. The total N content was 40 mg L^−1^ for each treatment solution. The contents of the other nutrients in all three treatment solutions were 10 mg P L^−1^, 40 mg K L^−1^, 40 mg Ca L^−1^, 40 mg Mg L^−1^, 5.6 mg Si L^−1^, 0.5 mg Mn L^−1^, 0.05 mg Mo L^−1^, 0.2 mg B L^−1^, 0.01 mg Zn L^−1^, 0.01 mg Cu L^−1^, and 2 mg Fe L^−1^. The nitrification inhibitor dicyandiamide was also added to each treatment solution at a concentration of 5 mg L^−1^. The experiments were conducted at an experimental site at Shenyang Agricultural University (41°48′ N, 123°25′ E) from April to October in 2016, and a rain shelter was used to cover the hydroponic pots of plants.

The plants of the three rice cultivars were cultured from seed to the four-leaf stage (35 days after sowing), and then three uniform plants were equidistantly transplanted into a plastic basket with a diameter of 30.0 cm. The roots were cleaned with demineralized water, and subsequently, each plastic basket was placed in a plastic pot (30.5 cm the uppermost diameter and 20.0 cm the lowest diameter, and 25.0 cm height) with 7 L of nutrient solution in the hydroponic system. Pots without plants were also set up to detect the effect of the hydroponic environment on N content. Six replicates were used for each treatment. All pots were arranged in a randomized design and re-randomized once every 10 days to minimize the position effects. The pH of the solutions was adjusted to 5.0 with either 1 M NaOH or 1 M HCl every day, while also adding an appropriate amount of demineralized water to supplement the loss by evapotranspiration. The nutrient solution was also replaced every 10 days. Three weeks before seed maturity, we replaced the culture solution with demineralized water (also at a pH of 5).

### Yield Components

Yield components including effective panicle number, seed-setting rate, 1,000-grain weight, grain number per panicle, and yield per plant were measured for each plant that was sampled. Filled and unfilled grains of the panicle were manually separated for measurements of the seed-setting rate. All filled grains from a single plant were collected and dried at 50°C for measurement of the grain yield per plant. Randomly selected filled grains were used for the 1,000-grain weight measurements.

### Biometric Parameters and N Content

After adding an appropriate amount of demineralized water to supplement the loss by evapotranspiration, the concentrations of $NH_{4}^{+}$ and $NO_{3}^{-}$ in the solutions were determined using a SEAL AutoAnalyzer 3 (Germany) every 10 days. Whole plants of SN265, SN1401, and SN9816 were sampled at the seed maturity stage. The plants were initially desiccated at 105°C, then oven dried at 80°C to a constant weight, and weighed. Biomasses of the leaves, stems (including sheaths), panicles, and roots were all measured prior to grinding to a power. The N concentrations of the leaves, stems (including sheaths), panicles, and roots were all determined by semi-micro Kjeldahl digestion and distillation (Nelson and Sommers, 1980).

We calculated the following indices from data collected from the dry matter weights, and N measurements, where the indices and their parameters are defined as follows: total N accumulation per plant (g plant^−1^) is the total amount of N accumulated in a plant after reaching seed maturity; N agronomic efficiency (kg kg^−1^) is grain yield divided by total N application; N recovery efficiency (%) is defined as total N accumulation divided by total N application for each rice plant; and N physiological efficiency (kg kg^−1^) is defined as grain yield divided by total N accumulation per rice plant.

### qRT-PCR and Enzyme Activity

The top leaves and roots of the rice plants were sampled to examine the differences in the expression of N metabolic genes and enzyme activity at the tillering stage (30 days after transplanting) and booting stage (the day that the top second leaf completely emerged after transplanting) (Wang et al., 2017). RNA extraction was carried out using the Eastep^®^ Super RNA extraction kit (Promega, Shanghai, China). Samples of 0.5 *μg* of total RNA were reverse transcribed into cDNA using the PrimeScript™ RT Master Mix kit (Takara, Dalian, China). Real-time quantitative RT-PCR (qRT-PCR) analysis was performed on cDNA using the TaKaRa SYBR^®^ Premix EX Ta kit and a 7500 Real-Time System (Applied Biosystems, USA). Rice ACTIN1 was used as the internal control in all analyses. The sequences of the gene-specific primers are shown in **Supplementary Table 2**.

The activity of NR was determined according to Gibon et al. (2004), GS according to Sun et al. (2014), NADH-GOGAT according to Singh and Srivastava (1986), and GDH was conducted according to Yamaya et al. (1984).

### Statistical Analysis

The data were statistically analyzed with Excel 2003 (Microsoft Office 2003) and SPSS22.0 for Windows (IBM Corporation), and means were tested by least significant difference at P <0.05 (LSD 0.05).

## Results

### Plant Architecture and Yield Components Under Different N Conditions

**Figure 1:**
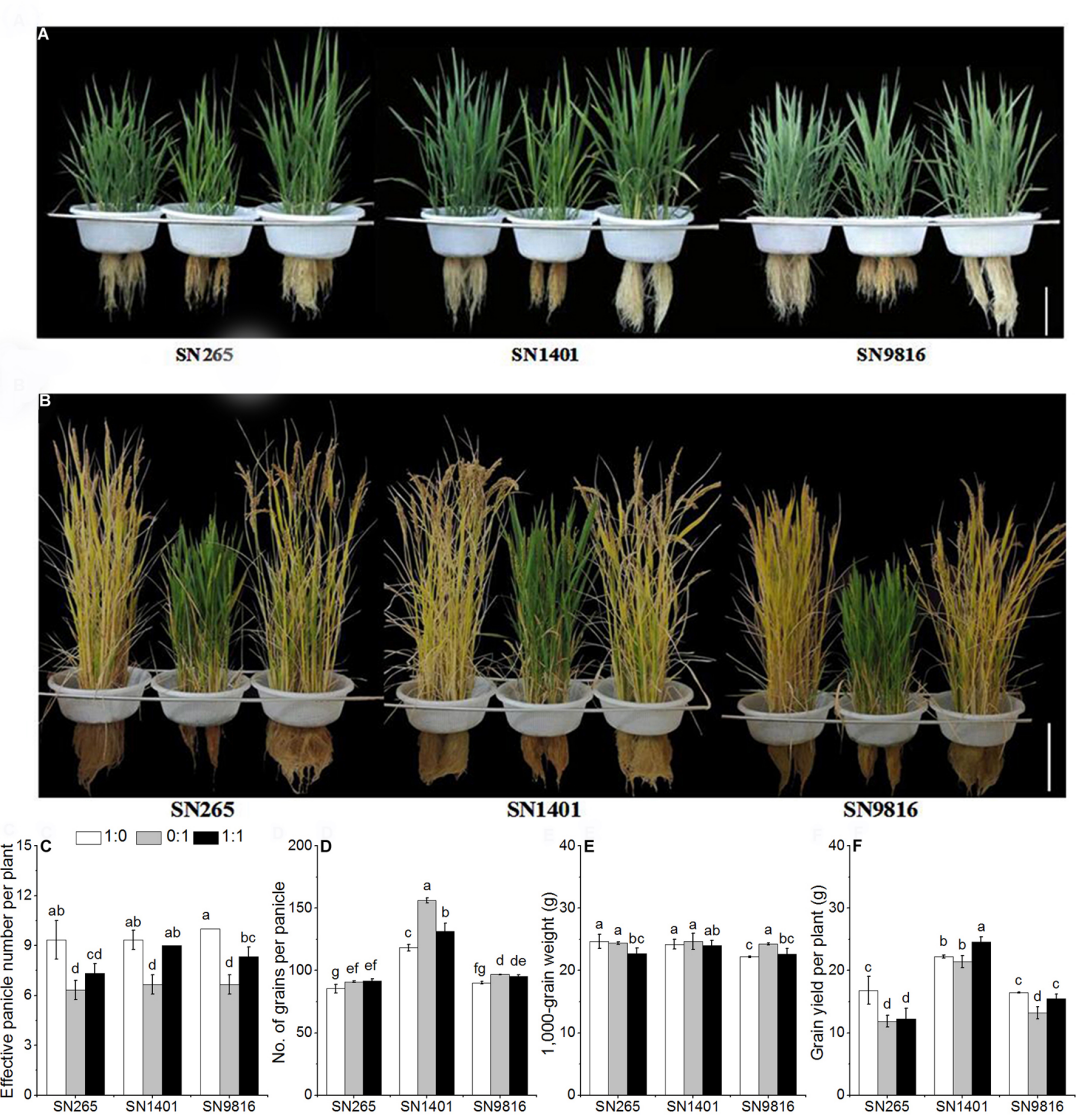
The plant architecture of three rice cultivars under different N nutrient solutions. **(A)** Rice plant architecture at 40 days after transplanting. **(B)** Rice plant architecture at 110 days after transplanting. The effects of N form on effective panicle number per plant **(C)**, number of grains per panicle **(D)**, 1,000-grain weight **(E)**, grain yield per plant **(F)** in three rice cultivars. 1:0, 0:1, and 1:1 were the proportions of $NH_{4}^{+}$:$NO_{3}^{-}$. Data were the means ± SE of six biological replicates. The presence of the same lower case letter above histogram bars denotes non-significant differences (P < 0.05). Scale bars, 20 cm.

In response to the different forms of N in the different treatments, the three *japonica* cultivars reached their maximum tillering stage approximately after 40 days. In the $NO_{3}^{-}$ nutrient solution, the life cycle of the *japonica* cultivars was longer than that of plants grown in the $NH_{4}^{+}$-sole solution, by about 25 days. Furthermore, plant growth and development exhibited a significant delay during the transition period from vegetative growth to that of reproductive growth (**Table 1**, **Figures 1A, B**).

**Table 1:** Growth and development stages of three *japonica* rice cultivars SN265, SN1401, and SN9816 expressed as days after transplanting.

Stage	$NH_{4}^{+}$:$NO_{3}^{—}$		
	1:0	0:1	1:1
Maximum tiller	40 b	55 a	40 b
Start of heading	69 b	86 a	68 b
Physiological maturity	120 b	145 a	120 b

No differences were observed between cultivars for each growth at developmental stages, and only significant differences were observed for the N form applied. Data were the means ± SE of six biological replicates. Different letters on each row indicate significant difference at P < 0.05.

With the treatment of only $NO_{3}^{-}$ in the solution, the effective panicle numbers per plant were significantly reduced. The mean effective panicle numbers of SN265, SN1401, and SN9816 were 32.3%, 27.9%, and 33.3% lower than the means of their respective cultivars grown in the $NH_{4}^{+}$-sole solution (**Figure 1C**). Overall, the numbers of grains per panicle in the $NO_{3}^{-}$ treatments were greater than those in the $NH_{4}^{+}$ treatments, especially in cultivar SN1401 (**Figure 1D**). Rice plants were significantly greater by 6.4%, 32.1%, and 7.3%, respectively, in SN265, SN1401, and SN9816. The 1,000-grain weight of the SN9816 seeds from the $NO_{3}^{-}$ treatment was significantly greater by 9.4% than that of the $NH_{4}^{+}$ treatment. However, the 1,000-grain weight of the SN265 and SN1401 seeds in the $NO_{3}^{-}$ treatment was not significantly different compared with those plants in the $NH_{4}^{+}$ treatment (**Figure 1E**). SN1401 showed the highest yields, regardless of the N treatment, with a better behavior under mixed nutrition. However, the SN265 and SN9816 plants grown in the $NO_{3}^{-}$ nutrient solution were significantly lower by 29.6% and 19.8%, respectively, compared with the plants grown in the $NH_{4}^{+}$ nutrient solution (**Figure 1F**).

### N Uptake and Utilization Under Different N Conditions

**Figure 2:**
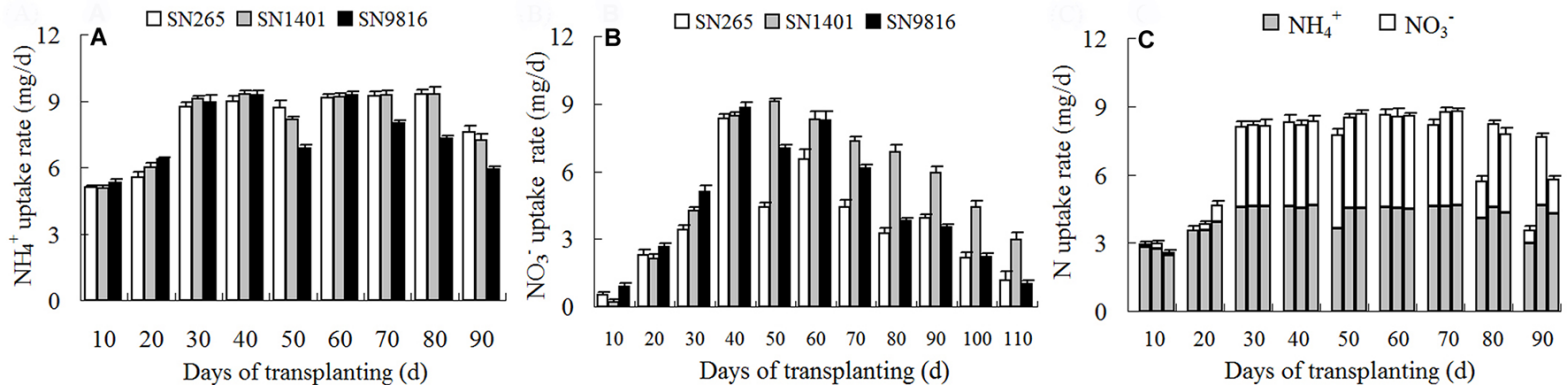
The N uptake rate of three rice cultivars under different N nutrient solutions. From transplanting to 3 weeks before seed maturity, the N uptake rate of rice plants under $NH_{4}^{+}$ **(A)**, $NO_{3}^{-}$ **(B)**, or $NH_{4}^{+}$ and $NO_{3}^{-}$ **(C)** nutrient solution every 10 days. **(C)** The three histograms every 10 days represented the N uptake rate of SN265, SN1401, and SN9816, respectively. Data were the means ± SE of six biological replicates.

For the entire duration of plant growth, the highest rates of N uptake in the rice were observed between 30 and 70 days after transplanting under the $NH_{4}^{+}$ treatments, which corresponded to the active tillering stage and to the end of the booting stage. However, the highest rates of N uptake in the $NO_{3}^{-}$-sole treatments were observed 40 to 60 days after transplanting, which corresponded to the maximum tillering stage and the middle of the booting stage. At the tillering stage (0–40 days after transplanting), the uptake rates of both $NH_{4}^{+}$ and $NO_{3}^{-}$ increased gradually; however, the uptake rate of $NH_{4}^{+}$ was clearly higher and peaked earlier than that of $NO_{3}^{-}$. Of the SN1401 plants, the uptake rate of $NH_{4}^{+}$ from the $NH_{4}^{+}$-sole solution remained at a high level between 60 and 80 days after transplanting, the days of which were consistent with the middle of the filling stage. After 80 days, the rate clearly began to decline. However, the uptake rate of $NH_{4}^{+}$ in the SN9816 plants began to rapidly decline after plant heading, about 70 days after transplanting (**Figure 2A**). Under the treatment with the $NO_{3}^{-}$ nutrient solution, the uptake rate of $NO_{3}^{-}$ increased gradually in all three rice plants, reaching their maximum values at 40 days after transplanting. The uptake rates of $NO_{3}^{-}$ at 60 days after transplanting, for the three rice plants, noticeably declined. However, the uptake rate of $NO_{3}^{-}$ declined slowly in SN1401 (**Figure 2B**). For the treatment with both $NH_{4}^{+}$ and $NO_{3}^{-}$, the N uptake rates began to decline 70 days after transplanting, and this was mainly due to the decrease in the uptake of $NO_{3}^{-}$. By 80 days after transplanting (the middle of the filling stage), the N uptake rate of the SN1401 plants was markedly higher than those of the SN265 and SN9816 plants (**Figure 2C**).

The total N taken by the rice plant was higher under the $NO_{3}^{-}$ -sole treatment because of not only the longer growth periods but also because the $NO_{3}^{-}$ uptake is still high after the booting stage. The total N accumulation of the SN1401 plants was significantly higher than that of the SN265 and SN9816 plants, growing in the $NO_{3}^{-}$ treatment and the treatment with both $NH_{4}^{+}$ and $NO_{3}^{-}$. We also established various indices indicative of N utilization by the plant. Overall, SN1401 was more efficient than SN265 and SN9816 in N use because the three indices of N use efficiency in SN1401 were higher than those of S265 and SN9816, under the same N treatments. Among the different N treatments, the N agronomic efficiencies of SN265, SN1401, and SN9816 plants in the $NO_{3}^{-}$ nutrient solution were significantly lower by 41.4%, 20.1%, and 33.3%, compared with those of the $NH_{4}^{+}$ solution, respectively. The N recovery efficiencies of SN265 and SN9816 under the $NO_{3}^{-}$ treatment were also significantly lower, by 37.3% and 31.2% than that of the $NH_{4}^{+}$ treatment, respectively. However, there were no differences in the N physiological efficiency of SN265 or SN9816 between the $NH_{4}^{+}$ and $NO_{3}^{-}$ treatments (**Table 2**).

**Table 2:** Nitrogen use efficiency of three *japonica* rice cultivars.

	$NH_{4}^{+}$:$NO_{3}^{—}$	Total N applied (g N per plant)	Total N accumulation (g N per plant)	N agronomic efficiency (kg·kg^−1^)	N recovery efficiency (%)	N physiological efficiency (kg·kg^−1^)
SN265	1:0	0.93	0.65 ± 0.01 d	18.1 ± 2.4 cd	70.3 ± 1.2 d	25.7 ± 3.0 c
	0:1	1.12	0.49 ± 0.00 g	10.6 ± 0.8 f	44.1 ± 0.3 g	24.0 ± 2.0 c
	1:1	0.93	0.63 ± 0.01 e	13.1 ± 1.8 e	67.8 ± 1.3 e	19.4 ± 2.4 d
SN1401	1:0	0.93	0.71 ± 0.01 b	23.9 ± 0.3 b	76.1 ± 0.7 a	31.4 ± 0.6 b
	0:1	1.12	0.83 ± 0.03 a	19.1 ± 0.9 c	74.5 ± 2.3 ab	25.7 ± 1.9 c
	1:1	0.93	0.71 ± 0.01 b	26.4± 0.8 a	76.0 ± 0.6 a	34.8 ± 0.9 a
SN9816	1:0	0.93	0.69 ± 0.02 bc	17.7 ± 0.1 cd	73.8 ± 1.9 bc	24.0 ± 0.7 c
	0:1	1.12	0.57 ± 0.00 f	11.8 ± 0.9 ef	50.8 ± 0.4 f	23.3 ± 1.9 c
	1:1	0.93	0.67 ± 0.01 cd	16.6 ± 0.8 d	72.3 ± 0.9 cd	23.0 ± 1.4 c

Data are the means ± SE of four biological replicates. Different letters indicate significant difference at P < 0.05.

### Relative Expression of N-Metabolizing Genes at Tillering and Booting Stages

**Figure 3:**
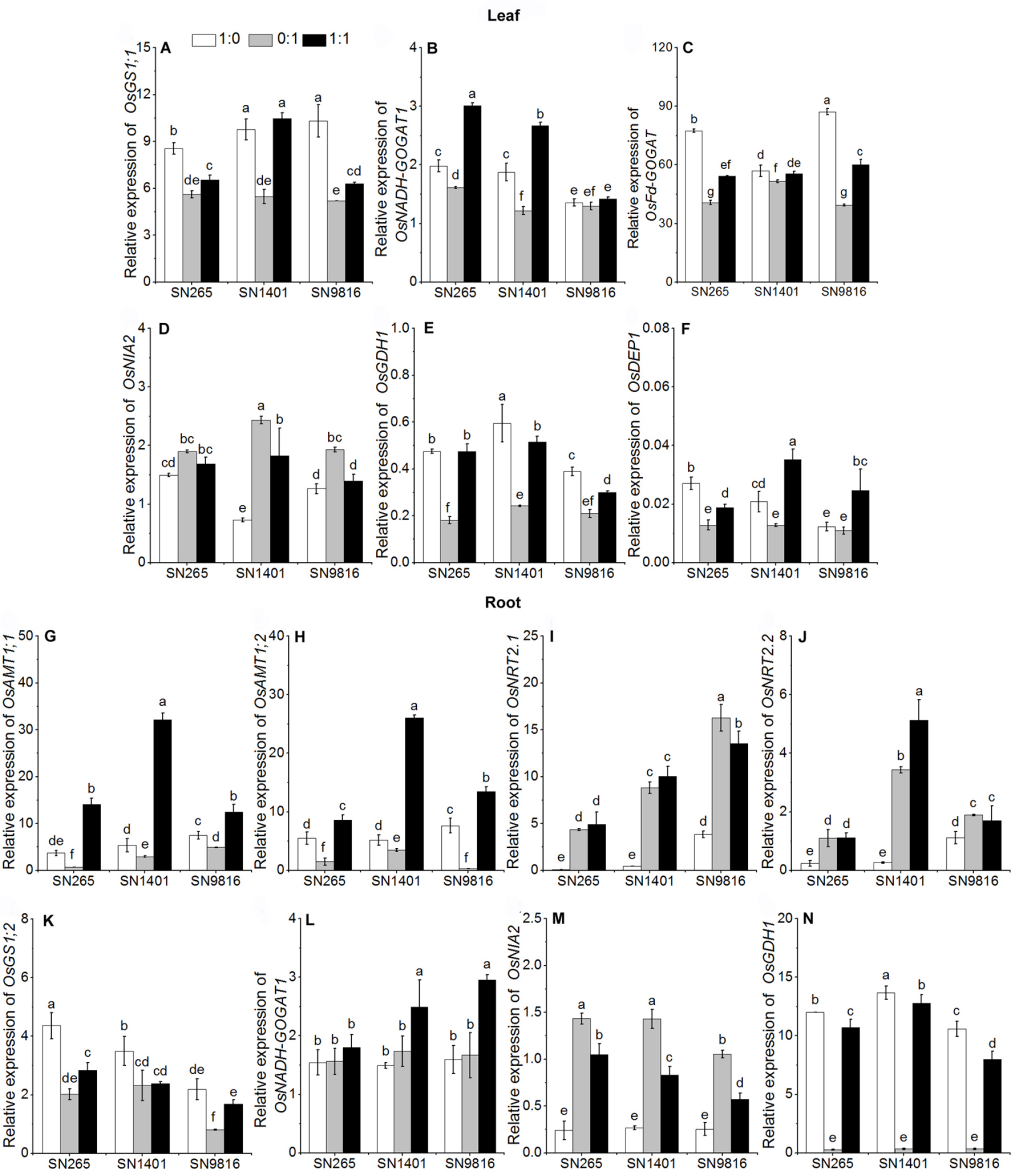
The transcription of genes involved in N uptake and assimilation in the leaves **(A**–**F)** and roots **(G**–**N)** at tillering stage. The proportions of $NH_{4}^{+}$:$NO_{3}^{-}$ were 1:0, 0:1, and 1:1. *DEP1*, *dense and erect panicle 1*, a gene that affects the numbers of grains per panicle and N use efficiency in the three rice cultivars. *Actin1* was used as internal standards. Data were the means ± SE of nine biological replicates. Different letters indicated significant difference at P < 0.05.

**Figure 4:**
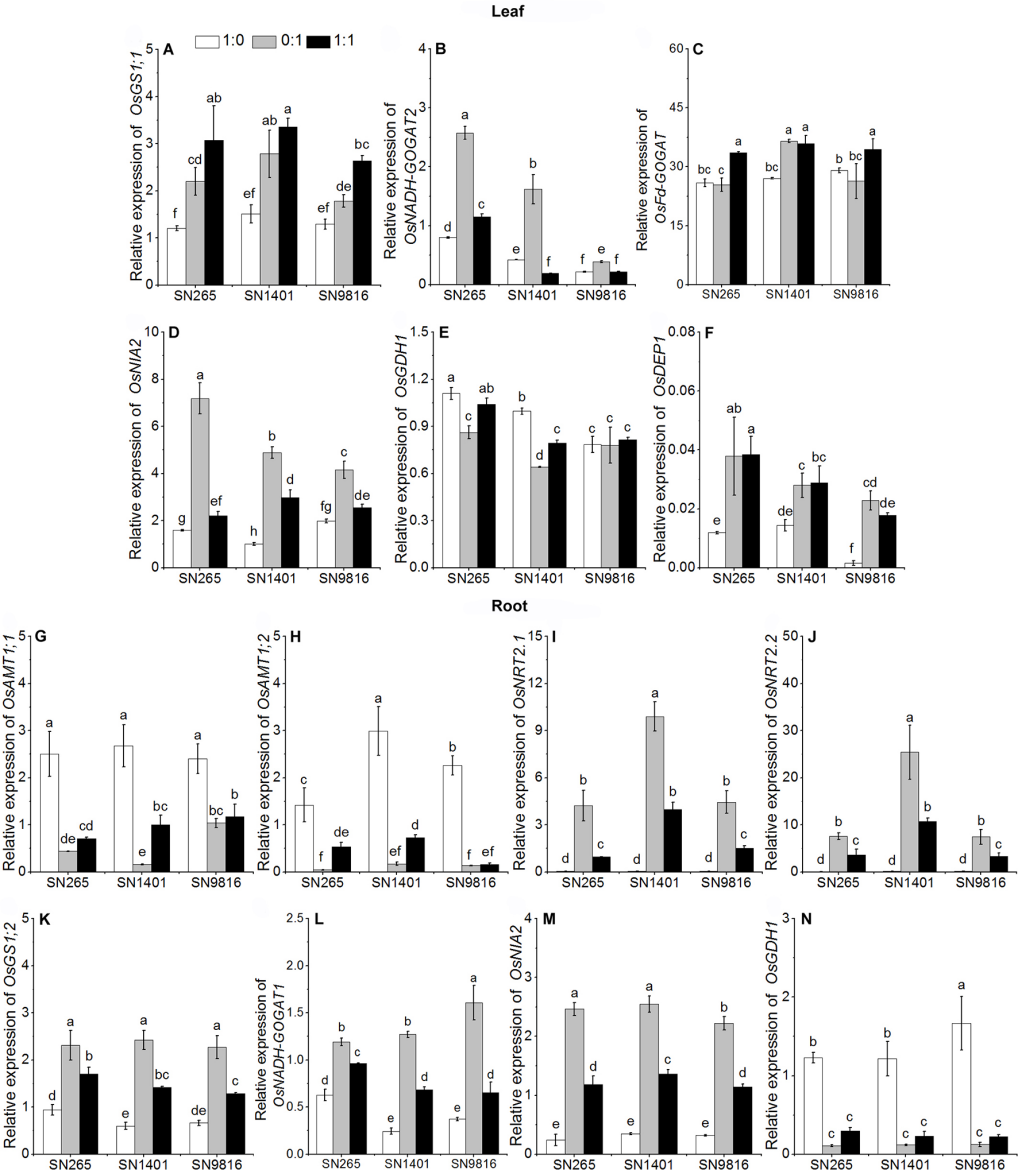
The transcription of genes involved in N uptake and assimilation in the leaves **(A**–**F)** and roots **(G**–**N)** at booting stage. The proportions of $NH_{4}^{+}$:$NO_{3}^{-}$ were 1:0, 0:1, and 1:1. *DEP1*, *dense and erect panicle 1*, a gene that affects the numbers of grains per panicle and N use efficiency in the three rice cultivars. *Actin1* was used as internal standards. Data were the means ± SE of nine biological replicates. Different letters indicated significant difference at P < 0.05.

The differences in grain yield per plant were mainly caused by the effective panicle number per plant and grain number per panicle, and considering that the active tillering and booting stages are the key stages for the formation of these two traits, respectively, we examined the differences in gene expression and enzyme activities involved in N uptake and those of N metabolism at the two stages (**Figures 3** and **4**). The results showed that at the tillering stage, the relative expression levels of *OsAMT1;1*, *OsAMT1;2*, and *OsNADH-GOGAT1* in the plant roots were highest under the treatment with both $NH_{4}^{+}$ and $NO_{3}^{-}$ (**Figures 3G**–**L**). The expression levels of *OsGS1;2* and *OsGDH1* in the roots of all three rice plants were highest under the $NH_{4}^{+}$ treatment (**Figures 3K, N**). In the leaves of the three rice plants, the relative expression levels of *OsGS1;1*, *OsFd-GOGAT*, and *OsGDH1* were higher with the $NH_{4}^{+}$-sole nutrient solution than with the $NO_{3}^{-}$-sole nutrient solution (**Figures 3A, C, E**). The relative expression level of *OsNADH-GOGAT1* was higher under the mixed nutrition (**Figure 3B**). In addition, because we observed differences in grain counts, we also examined the expression levels of *OsDEP1* (a gene that affects the numbers of grains per panicle and N use efficiency in the three rice cultivars) and determined that in SN265, SN1401, and SN9816, the expression levels in the plant leaves were lowest under the $NO_{3}^{-}$-sole treatment at the tillering stage (**Figure 3F**).

At the booting stage, the *OsAMT1;1* and *OsAMT1;2* in the plant roots were more expressed under the $NH_{4}^{+}$ treatment and mixed nutrition, especially in SN1401 (**Figures 4G, H**). The relative expression levels of the nitrate transporter genes, *OsNRT2.1* and *OsNRT2.2*, in the SN1401 were also significantly higher than those for the SN265 and SN9816 roots grown in the $NO_{3}^{-}$-sole or $NH_{4}^{+}$ and $NO_{3}^{-}$ nutrient solutions (**Figures 4I, J**). Under the $NO_{3}^{-}$ treatment, the expression levels of the nitrate reductase gene *OsNIA2* in SN9816 was significantly lower than in SN265 and SN9816 (**Figure 4M**). The expression levels of *OsGS1;2* and *OsGDH1* in the plant roots were higher under the $NH_{4}^{+}$-sole treatment (**Figures 4K, N**). The expression levels of *OsNADH-GOGAT1* in the roots were highest in the treatment with both $NH_{4}^{+}$ and $NO_{3}^{-}$ (**Figure 4L**). The expression levels of *OsGS1;1* and *OsFd-GOGAT* in the leaves of all three rice plants were also highest in the treatment with both $NH_{4}^{+}$ and $NO_{3}^{-}$ (**Figures 4A, C**). The expression levels of *OsNADH-GOGAT2* and *OsNIA2* in the leaves were significantly the highest in the $NO_{3}^{-}$ treatment (**Figures 4B, D**). The expression levels of *OsGS1;1*, and *OsFd-GOGAT* in the SN1401 leaves were significantly higher than those of the SN265 and SN9816 plants in the $NO_{3}^{-}$ solution. The expression levels of *OsDEP1* were lower under the $NH_{4}^{+}$-sole treatment than with the other two treatments (**Figure 4F**).

### N Metabolism Enzyme Activities at Tillering and Booting Stages

**Figure 5:**
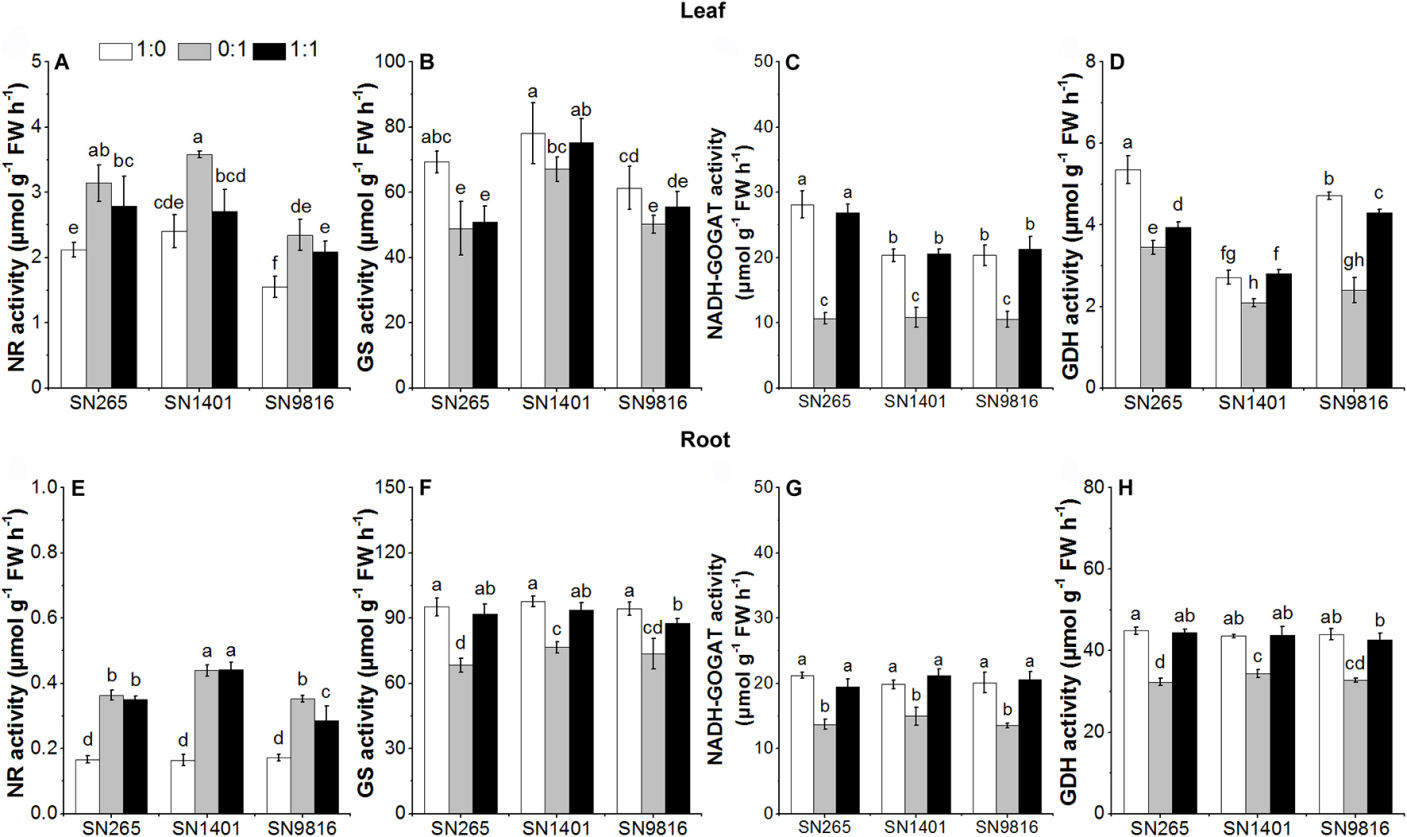
The activity of enzymes involved in N metabolism in the leaves **(A**–**D)** and roots **(E**–**H)** at tillering stage. The proportions of $NH_{4}^{+}$:$NO_{3}^{-}$ were 1:0, 0:1, and 1:1. Data were the means ± SE of six biological replicates. Different letters indicated significant difference at P < 0.05.

At the tillering stage, the activity levels of GS, NADH-GOGAT, and GDH in the roots and leaves of the three rice plants in the $NH_{4}^{+}$ treatment were significantly higher than those of the plants with the $NO_{3}^{-}$ treatment. Among the rice cultivars, the activities of NR and GS in roots and leaves of SN1401 were significantly higher than those of SN265 and SN9816 in the $NO_{3}^{-}$ treatment (**Figure 5**).

**Figure 6:**
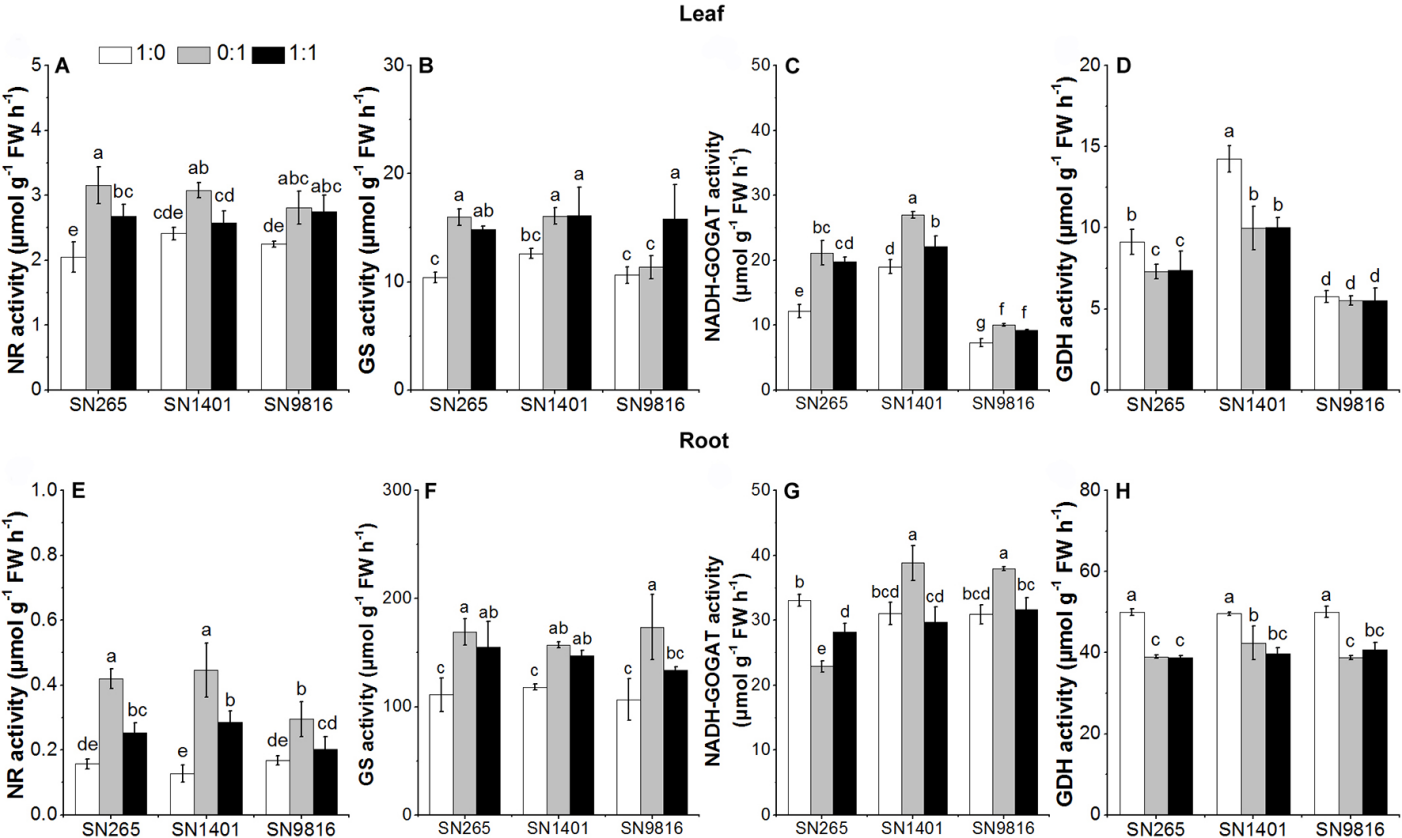
The activity of enzymes involved in N metabolism in the leaves **(A**–**D)** and roots **(E**–**H)** at booting stage. The proportions of $NH_{4}^{+}$:$NO_{3}^{-}$ were 1:0, 0:1, and 1:1. Data were the means ± SE of six biological replicates. Different letters indicated significant difference at P < 0.05.

At the booting stage, the activities of NR and GS in the roots and leaves of the three rice plants showed lower enzymatic activities in the $NH_{4}^{+}$-sole treatment, and overall minimal differences were observed among the cultivars for each N treatment. On the other side, the root GDH activity was higher under the $NH_{4}^{+}$-sole nutrition. The NADH-GOGAT and GDH activities were significantly higher in the leaves of SN1401 in all N treatments (**Figure 6**).

## Discussion

N is an essential element for plant growth and development and is an important factor affecting rice growth and yield (Vinod and Heuer, 2012), and subsequently, improving N use efficiency is of key importance (Peng et al., 2010). In this study, we observed that after transplanting of the rice seedling, the uptake rate of $NH_{4}^{+}$ was faster than the uptake rate of $NO_{3}^{-}$ in the tillering stage (**Figure 2**). Besides, the $NH_{4}^{+}$ uptake is maintained until the end of the booting stage. Lower uptake rates of $NO_{3}^{-}$ in the $NO_{3}^{-}$ treatment may affect the uptake and utilization of other nutrient elements such as phosphorus (Fan et al., 2016) and iron (Zhu et al., 2016) and, thus, affect protein synthesis and metabolism in plants. In turn, plant growth and development will be affected, potentially resulting in prolonged growth periods (**Figure 1**, **Table 2**). However, the underlying mechanisms of the prolonged growth periods affected by $NO_{3}^{-}$ need further study. In addition, there were almost no change in the concentration of $NH_{4}^{+}$ or $NO_{3}^{-}$ in the pots without plants, indicating that the environment had little effect on the concentration of $NH_{4}^{+}$ and $NO_{3}^{-}$ in the experiments. Lian et al. (2012) investigated the kinetic uptake of $NH_{4}^{+}$ and $NO_{3}^{-}$ from NH_4_NO_3_ nutrient solutions in 23 *indica* rice cultivars from all over the world and found that the maximum uptake rates of $NH_{4}^{+}$ were significantly higher than those of $NO_{3}^{-}$ at both 10 mg L^−1^ and 40 mg L^−1^ N concentrations. In our study, we found that all three rice cultivars preferred to uptake $NH_{4}^{+}$ rather than $NO_{3}^{-}$, and the uptake rates of $NO_{3}^{-}$ were low in all three cultivars at the tillering stage, and the uptake rate of N began to decline at 60–70 days after plants transplanting, especially the significant decline in the uptake rate of $NO_{3}^{-}$. Furthermore, under the 1:1 $NH_{4}^{+}$:$NO_{3}^{-}$ treatment, the decrease in N uptake by rice plants was mainly due to the lower uptake rate of $NO_{3}^{-}$ (**Figure 2**). We also found for plants at the booting stage, grown in the $NO_{3}^{-}$ nutrient solution, that the expression levels of *OsNRT2.1* and *OsNRT2.2* in SN1401 roots and the activity of NADH-GOGAT in SN1401 were higher than those of SN265 and SN9816, and this corresponded to the higher N uptake rates of SN1401, compared with SN265 and SN9816. The higher $NO_{3}^{-}$ uptake rates of SN1401 at the reproductive growth stage may also result in higher N recovery efficiencies of SN1401 compared with those of SN265 and SN9816. Youngdahl et al. (1982) and Hu et al (2015) also found that N uptake rates were significantly lower at the seedling and tillering stages of rice plants grown in the $NO_{3}^{-}$-sole solution, which resulted in lower dry matter and tiller numbers. The photosynthetic rates of the rice leaves were improved by topdressing with $NO_{3}^{-}$ at the later growth stage, and the grain yields of rice with high N use efficiency were also improved by partial nitrate nutrition (Luo et al., 1993; Duan et al., 2007). Therefore, according to the N uptake characteristics of the different types of rice at every stage, empirically based adjustments to N application could effectively increase the uptake of $NO_{3}^{-}$ at later stages of plant growth, which ultimately will be beneficial for increasing N utilization and the rice yields.

As excessive $NH_{4}^{+}$ in cells may cause plant poisoning, most ions of $NH_{4}^{+}$ are assimilated in the root. However, $NO_{3}^{-}$ is usually transported from root to the mesophyll cells for its reduction, and the remaining $NO_{3}^{-}$ ions are reduced in root cells and stored in the vacuoles (Tegeder and Masclaux-Daubresse, 2018). Due to the differences in N uptake of the rice grown in culture with different forms of N, the expression of related genes involved in N metabolism in leaves and roots significantly differed (Gaur et al., 2012). $NH_{4}^{+}$ assimilation mainly occurs through synergistic catalysis of GOGAT, Fd-GOGAT, and NADH-GOGAT (Yang et al., 2016). *OsFd-GOGAT* is mainly expressed in the plastids of mesophyll cells and is responsible for the $NH_{4}^{+}$ re-assimilation coming from $NO_{3}^{-}$ reduction and photorespiration (Chen et al., 2016). Especially, there are two types of *OsNADH-GOGAT*: (1) *OsNADH-GOGAT1* is primarily distributed in the root epidermis and cortex cells and participates with *OsGS1;2* in the assimilation of $NH_{4}^{+}$ in roots; and (2) *OsNADH-GOGAT2* is primarily expressed in the vascular bundles of mature leaves (Ma et al., 2016). Moreover, the *OsGS1;2* and *OsNADH-GOGAT1* are important in the development of active tiller number (Tamura et al., 2010; Funayama et al., 2013), and the *OsGS1;1* and *OsNADH-GOGAT2* also play an important role in rice grain development (Tamura et al., 2011). In our results, the activities of GS and NADH-GOGAT of the three rice plants were higher when $NH_{4}^{+}$ was present (either as sole N source or as mixed nutrition) at the tillering stage, and with the $NO_{3}^{-}$ treatment (both applied as sole N source and also as mixed nutrition) at the booting stage. This result suggests that the effects of N form on the primary assimilation of $NH_{4}^{+}$ in the three rice cultivars are different at different stages of plant growth (Lam et al., 1996). At the tillering stage, the expression level of *OsGS1;2* and *OsGDH1* and the activity of GS, NADH-GOGAT, and GDH in the roots, and the expression levels of *OsGS1;1*, *OsFd-GOGAT*, and *OsGDH1*, and the activity of NADH-GOGAT and GDH in the leaves were higher when $NH_{4}^{+}$ was present (either as sole N source or as mixed nutrition) than those of the plants with the $NO_{3}^{-}$-sole treatment. The higher gene expression levels and enzyme activities increased N assimilation, which also promoted the rice plants growth and development with the $NH_{4}^{+}$ treatment at the tillering stage. We also found that in the $NH_{4}^{+}$ treatment, the expression levels of *OsGS1;1* or *OsFd-GOGAT* in the leaves were not significantly different among the three rice cultivars at the booting stage. However, with the $NO_{3}^{-}$ treatment, the expression levels of *OsGS1;1* and the activity of GS, NADH-GOGAT, and GDH in the SN1401 leaves were significantly higher than those of SN265 and SN9816 at the booting stage. Those results showed that the difference in expression of genes controlling N-metabolism enzymes in relation to the genetic background of the cultivars (Gaur et al., 2012; Li et al., 2017), and SN1401 had a better capacity in N assimilation at the booting stage, which promoted the panicle development of SN1401. The higher expression levels of *OsFd-GOGAT* and activities of GS in the leaves may also represent that SN1401 had a better capacity in secondary assimilation of $NH_{4}^{+}$ at the booting stage (Yamaya and Kusano, 2014). However, this higher secondary assimilation ability of SN1401 still needs to be confirmed by further testing the expression level of *GS2* and the activity of Fd-GOGAT in the leaves at booting stage.

In addition, some researchers found that plants differed in N utilization due to the differences in genotypes (Song et al., 2011). Koutroubas and Ntanos (2003) found that the N harvest index (the proportion of N content in the grain in relation to total N of the whole plant) of *japonica* varieties was lower than those of *indica* varieties, which suggests that N physiological efficiencies were also lower. Qian et al. (2004) found that the 1,000-grain weight of the *indica* cultivar Shanyou63 was significantly greater after topdressing with $NO_{3}^{-}$ than with $NH_{4}^{+}$. Moreover, the N content of grains was also higher with the $NO_{3}^{-}$ topdressing. The yield of rice is mainly determined by four components: the number of effective panicles per area, the number of grains per panicle, the seed setting rate, and the grain weight (Li et al., 2013). In this study, we found that under the $NO_{3}^{-}$-sole condition, the number of grains per panicle of SN1401 was significantly greater than for those of the two other treatments. Although fewer tillers were produced during the vegetative growth stage (most likely because of the lower $NO_{3}^{-}$ uptake rate), SN1401 plants were able to produce a higher number of grains per panicle, thus compensating the decrease in the effective panicle number per plant and showing even higher grain yield per plant than SN265 and SN9816 plants. The N agronomic efficiency and N recovery efficiency of SN1401 were also significantly higher than those of SN265 and SN9816. Therefore, our results of the differences in N uptake and utilization characteristics between the three rice cultivars given varying forms of N are applicable for improving agricultural use of N fertilizer in rice production. Moreover, according to the effects of the different forms of N on the effective panicle number and grain number per panicle, the N fertilizer application and irrigation water management could be regulated in the field (Yao et al., 2012; Sun et al., 2012), to reduce the conversion of $NH_{4}^{+}$ to $NO_{3}^{-}$ in the rhizosphere of rice plants, thereby increasing the $NH_{4}^{+}$ uptake of rice plants in the vegetative growth stage; and to increase the content of $NO_{3}^{-}$ in the rhizosphere of rice plants, thereby increasing the $NO_{3}^{-}$ uptake of rice plants in the reproductive growth stage, and finally to improve rice yield and nitrogen utilization.

## 

## Author Contributions

JY conducted all the experiments. JG and WZ designed the experiments and edited the manuscript. CZ and XZ measured the gene expression. YW was involved in nitrogen measurement.

## Funding

This research was supported by the National Key R&D Program (2018YFD0300306), Chinese Natural Sciences Foundation (31501250), Liaoning BaiQianWan Talents Program (2015-39), and Shenyang Science and Technology Plan Project (17-231-1-37).

## Conflict of Interest Statement

The authors declare that the research was conducted in the absence of any commercial or financial relationships that could be construed as a potential conflict of interest.
