# Supplementary material for: Differential Uptake and Utilization of Two Forms of Nitrogen in Japonica Rice Cultivars From North-Eastern China
Source: Front Plant Sci. 2019 Sep 4;10:1061. doi: 10.3389/fpls.2019.01061 (PMC6738331; doi:10.3389/fpls.2019.01061)
Supplement: Supplementary file 1 [file Table_1.doc]

Supplementary Material

# Differential uptake and utilization of two forms of nitrogen in japonica rice of northeastern China

**Jun Yi, Jiping Gao, Wenzhong Zhang*, Chen Zhao, Yan Wang and Xiaoxi Zhen**

*** Correspondence:** wenzhong Zhang: zwzhong@126.com

# Supplementary Table

**Table 1 Agronomic traits of three *japonica* rice cultivars grown in field (225 kg N ha^−1^) in north-eastern China in 2015.**

| Cultivars | Effective panicle number per plant | No. of grains per panicle | Seed setting rate  (%) | 1,000-grain weight  (g) | Grain yield  (t ha^−1^) |
| --- | --- | --- | --- | --- | --- |
| SN265 | 15.1 a | 130.1 b | 85.5 a | 24.1 a | 9.2 b |
| SN1401 | 14.6 a | 153.8 a | 80.2 b | 24.6 a | 10.3 a |
| SN9816 | 14.5 a | 139.1 b | 83.1 ab | 23.6 a | 9.5 b |

Data were the means ± SE of six biological replicates. Different letters on each column indicates signiﬁcant difference at P < 0.05.

**Table 2** Primer sequences used for quantitative real-time PCR.

| Gene name | Genebank ID | Forward sequence | Reverse sequence |
| --- | --- | --- | --- |
| OsAMT1;1 | AF289477.1 | ggtttctctccctctccgat | ccaccttcacaccacacatt |
| OsAMT1;2 | AF289478.1 | aagcacatgccgcagaca | gacgcccgacttgaacag |
| OsNRT2.1 | AB008519.1 | cttcacgtcgtcgaggtact | cactcggagccgtagtagtg |
| OsNRT2.2 | AK109733.1 | catcgccgagtacttctac | atccaaatgttccagaggcg |
| OsGS1;1 | AB037595.1 | caccaacaagaggcacaatg | actcccactgtcctggcat |
| OsGS1;2 | AB180688.1 | tgtttctcctcatccctgc | tcacagtcctcgctttgc |
| OsNADH-GOGAT1 | AB008845.1 | gtgcagcctgttgcagcataaa | cggcatttcaccatgcaaatc |
| OsNADH-GOGAT2 | AB274818.1 | cctgtcgaaggatgatgaaggtgaaacc | tgcatggccctactatcttcgcatca |
| OsFd-GOGAT | Y12594.1 | gcatacttgtgaagcaccgaagtg | ctgcaaatagcaacctagcgtcag |
| OsGDH1 | BAE48296.1 | catctgatcatctccctgtt | ttcaggcaattcatcactac |
| OsNIA2 | BAF23923.1 | tgtaccaggtcatccagtcg | cgatgacgtaccacaccttg |
| OsDEP1 | FJ039905.1 | gcgagatcacgttcctcaag | tgcagtttggcttacagcat |
| OsACTIN1 | AB047313.1 | accattggtgctgagcgttt | cgcagcttccattcctatgaa |
